# Trends in the mobility of primary healthcare human resources in underdeveloped regions of western China from 2000 to 2021: Evidence from Nanning

**DOI:** 10.1186/s12875-024-02403-7

**Published:** 2024-05-06

**Authors:** Xinyi Xu, Jingyi Huang, Xiaoqian Zhao, Yumin Luo, Linxuan Wang, Yishan Ge, Xingyin Yu, Pinghua Zhu

**Affiliations:** School of Humanities and Social Sciences, Guangxi Medical University, Nanning, China

**Keywords:** China, Primary healthcare personnel, Healthcare reform, Primary health care

## Abstract

**Objective:**

This research aimed to identify the fundamental and geographic characteristics of the primary healthcare personnel mobility in Nanning from 2000 to 2021 and clarify the determinants that affect their transition to non-primary healthcare institutions.

**Methods:**

Through utilizing the Primary Healthcare Personnel Database (PHPD) for 2000–2021, the study conducts descriptive statistical analysis on demographic, economic, and professional aspects of healthcare personnel mobility across healthcare reform phases. Geographic Information Systems (QGIS) were used to map mobility patterns, and R software was employed to calculate spatial autocorrelation (Moran’s I). Logistic regression identified factors that influenced the transition to non-primary institutions.

**Results:**

Primary healthcare personnel mobility is divided into four phases: initial (2000–2008), turning point (2009–2011), rapid development (2012–2020), and decline (2021). The rapid development stage saw increased mobility with no spatial clustering in inflow and outflow. From 2016 to 2020, primary healthcare worker mobility reached its peak, in which the most significant movement occurred between township health centers and other institutions. Aside from their transition to primary medical institutions, the primary movement of grassroots health personnel predominantly directs towards secondary general hospitals, tertiary general hospitals, and secondary specialized hospitals. Since 2012, the number and mobility distance of primary healthcare workers have become noticeably larger and remained at a higher level from 2016 to 2020. The main migration of primary healthcare personnel occurred in their districts (counties). Key transition factors include gender, education, ethnicity, professional category, general practice registration, and administrative division.

**Conclusions:**

This study provides evidence of the features of primary healthcare personnel mobility in the less developed western regions of China, in which Nanning was taken as a case study. It uncovers the factors that impact the flow of primary healthcare personnel to non-primary healthcare institutions. These findings are helpful to policy refinement and support the retention of primary healthcare workers.

**Table:** 

Contributions to the literature
• Utilize big data to discover the influencing factors of grassroots health personnel moving to non-primary healthcare institutions.
• Most of the current literature describes the mobility of grassroots health personnel in developed provinces of China, with less research on the mobility situation of primary healthcare workers in less developed provinces in Western China.
• Investigated the temporal and spatial characteristics of the mobility of primary healthcare workers in Western Chinese cities.

## Introduction

The attrition of primary healthcare physicians has appeared as a focal point of international research [1–4]. In China, primary healthcare workers, the principal bearers of China’s first-contact care system, play a pivotal role in constructing a tiered diagnosis and treatment system, which underpins China’s medical and health enterprises. Since the 21st century, China has issued a myriad of policies to fortify its grassroots healthcare workforce. Initially, the nation underscored the imperative of enhancing the educational and training programs for primary healthcare personnel in 2009. After 2015, grassroots medical and health institutions across China collaborated with higher-level medical entities to pioneer the establishment of medical alliances [5]. To entice these healthcare professionals to remain at the grassroots level, diverse regions in China have improved their salary and welfare packages. In terms of personnel management, an integrated service management approach is adopted for rural doctors, which gives rise to a novel management paradigm that features unification in administration, personnel, operations, medical equipment assets, performance evaluation, and finance. Additionally, China implemented a policy of rural-targeted tuition-free medical education [6]. Between 2010 and 2020, the nation cultivated over 70,000 medical students under this rural-oriented tuition-free program, amounting to an average of 1.9 undergraduate students directed towards each township health centre in the central and western regions.

In the western regions of China, densely populated grassroots communities demonstrate a huge demand for primary healthcare services. Following the recent healthcare reforms, prominent hospitals have exerted a marked ‘siphoning’ impact on grassroots medical and health personnel. Concurrently, there is a discernible level of dissatisfaction in job roles among these primary healthcare providers, evidenced by a notable incidence of professional burnout [7–11]. This has further intensified the attrition of primary healthcare staff. Moreover, the service quality offered by these professionals appears to be below optimal [12]. Therefore, it becomes imperative to explore strategies and design robust health policies to mitigate this staff loss and entice many medical practitioners to serve at the grassroots level.

Nevertheless, previous studies are predominantly reliant on sample surveys [10]. There remains a void in research that explores the large-scale data on the mobility of Chinese primary healthcare personnel over extended periods. In addition, after the healthcare reform in 2009, there is quite limited research that addresses the mobility trends of these grassroots healthcare professionals. While previous research has examined the mobility rate and influencing factors of primary healthcare physicians in the U.S., there is a discernible gap in the literature that analyzes their mobility from both temporal and geographical perspectives [13].

Nanning City in the Guangxi Zhuang Autonomous Region, situated in the less-developed minority areas of western China, boasts a significant number of primary healthcare workers, which makes its trends in healthcare human resource mobility noteworthy. This study utilizes data from 2000 to 2021 on the movement of primary healthcare personnel in Nanning. The target is to discern the fundamental characteristics, temporal and spatial attributes, directional preferences, and principal influencing factors of human resource mobility in primary healthcare in underdeveloped regions of western China during diverse phases of healthcare reform. Insights drawn can be utilized to inform and enhance policymaking related to the improvement of the grassroots healthcare workforce.

## Method

### Sample and research design

China’s healthcare reform in the 21st century has undergone multiple phases, with scholars dividing its stages in varying ways. For instance, Tao et al. broadly categorized the reform into three primary phases [14]. To be specific, the first phase spans the first three decades following the establishment of the People’s Republic of China, from 1949 to 1979. The next phase encompasses the three decades after the economic reforms, from 1979 to 2009. The most recent phase termed the new healthcare reform, extends from 2009 to 2020, and is further separated into three sub-phases: The 2009–2011 phase, The 2012–2015 phase, and the 2016–2020 phase. In contrast, Yip et al. divided the post-2009 new healthcare reform into two segments [15]: 2009–2011 and 2012–2019. Drawing from the existing literature, this research classifies the healthcare reform post-2000 into five stages: 2000–2008, 2009–2011, 2012–2015, 2016–2020, and 2021 onwards. The primary sources for the policies of primary healthcare human resources in Nanning City across the five stages were derived from official policy documents publicly available on the websites of the Nanning City Health Commission and the Guangxi Zhuang Autonomous Region Health Commission.

The primary healthcare personnel database (PHPD) for Nanning City spans from 2000 to 2021. This database is bifurcated: the demographic and professional attributes of the inflow and outflow personnel (DPAP) and specific migration patterns of the outgoing personnel (SMPP). The influx and outflux characteristics of personnel across grassroots healthcare institutions mainly comprise data from four types of institutions: community health service center (CHSC), community health service station (CHSS), township health center (THC), and village clinic (VC). In the second section of the database, primary healthcare institutions do not cover village health clinics. Medical staff in these primary healthcare entities encompasses various professional categories, including nurses, practicing physicians, and administrative personnel. This dataset encapsulates fundamental details, such as gender, ethnicity, profession, and highest educational attainment of all incoming and outgoing primary healthcare staff. Moreover, for those exiting, the second section of the dataset not only presents basic attributes but also offers information on their originating institution, destination institution and year of migration. This study identified the longitude and latitude of each institution in the database to examine the spatial characteristics of the data. Also, it extracted data from the ‘Nanning City Statistical Yearbook, which encompassed such metrics as per capita GDP, degree of urbanization, permanent resident population, fiscal expenditure on health care, educational funding, and average wages in the health sector for all districts and counties of Nanning City from 2016 to 2020. This intends to clarify the spatial concentration of the movement of primary healthcare personnel in Nanning City and the associated influencing factors.

### Measures and analysis

To begin with, this study selected descriptive data on the inflow and outflow of primary healthcare personnel in Nanning City and performed a descriptive statistical analysis of their characteristics. By utilizing the longitudinal and latitudinal data of each institution, QGIS was adopted to plot the heatmaps revealing the inflow and outflow points of primary healthcare personnel across the five phases. In addition, this research also applied the Yuill method and charted the centroid of movement and the standard deviational ellipse for personnel across different phases.

Secondly, spatial correlation analysis of primary healthcare personnel mobility was conducted using the R programming language. With the ‘spdep’ package, the construction of a spatial weight matrix constitutes a fundamental procedure for examining spatial correlations [16]. Given the fact that the movement of primary healthcare workers in various regions is affected by the economic conditions of those regions, with regional GDP per capita being a crucial indicator, this research constructed a geographical economic matrix based on regional GDP per capita using R. Upon successfully constructing the matrix, it proceeded with spatial correlation analysis. The primary tool for this analysis was the global Moran’s I. Moran’s I values range between [-1,1]. If the p-value of Moran’s I is below 0.05, then there exists a significant difference between regions. If Moran’s I is in [0,1], then it reveals positive spatial autocorrelation, meaning similar attributes cluster together. Conversely, a Moran’s I value in [-1,0] denotes negative spatial autocorrelation, which suggests dissimilar attributes cluster together. If Moran’s I is 0, then attribute values are randomly distributed in space [17].

Thirdly, this research analyzed the specific flow data of primary healthcare personnel. Through the use of R, it generated Sankey diagrams and utilized the ‘pheatmap’ package to create heatmaps [18], thereby elucidating the principal categories of institutions receiving primary healthcare workers and quantifying the magnitude of their transitions. With QGIS, migration maps of primary healthcare personnel were developed and a descriptive analysis was carried out on the top 10 primary healthcare institutions in terms of movement, which unveiled spatial characteristics.

**Fig. 1:**
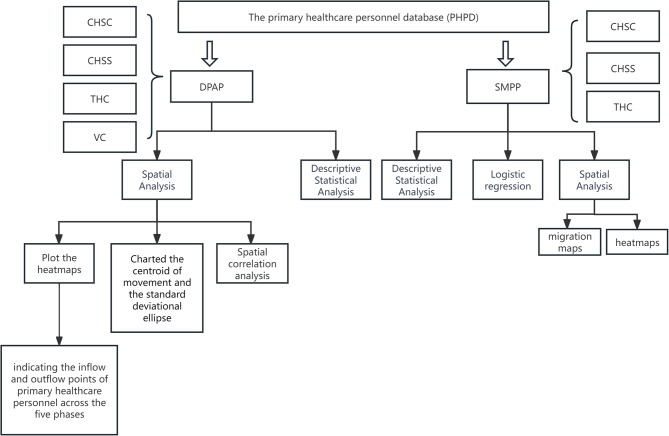
Data processing flowchart

Finally, this research filtered out all primary healthcare personnel who moved to non-primary healthcare institutions. Logistic regression was performed to identify the personal characteristics that influence their movement decision (See Fig. 1).

## Result

### Basic characteristics of primary healthcare personnel mobility

#### Community health service stations (CHSS)

A total of 919 primary healthcare personnel migrated into Nanning’s CHSSs. In terms of gender, 21.88% were male while the remaining 78.13% were female. Registered nurses accounted for 42.11%, while practicing physicians made up 33.30%. Notably, 97.28% of these healthcare workers had not acquired the national standardized training qualification certificate for resident physicians. In terms of professional technical positions, 35.47% were at the teacher level, 33.73% at the scholar level, and 18.61% at the intermediate level. From the perspective of educational qualifications, 47.23% had associate degrees or vocational school degrees, 23.50% had secondary vocational qualifications, and 26.12% held bachelor’s degrees. Moreover, 84.22% were not registered in general medicine, and 43.42% had not practiced in multiple locations. Their movement was motivated by varying aspects, with 55.39% due to other reasons and 38.41% transferring from other health institutions.

Regarding outflow, 368 primary healthcare workers left the CHSSs, with a gender distribution of 23.54% male and 76.46% female. Registered nurses and practicing physicians comprised 40.21% and 29.63%, respectively. A significant 98.41% had not acquired the national standardized training certificate for resident physicians. For professional roles, 38.62% were at the scholar level and 28.31% at the teacher level. In terms of academic credentials, 42.06% had associate or vocational school qualifications, 32.01% had secondary vocational credentials, and 21.16% possessed bachelor’s degrees. Furthermore, 88.89% were not registered in the field of general medicine, and 49.47% did not practice across multiple locations. The major motivations for their departure covered resignation or dismissal at 49.74%, transfers to other health institutions at 26.46%, and other reasons accounting for 20.11%.

**Fig. 2:**
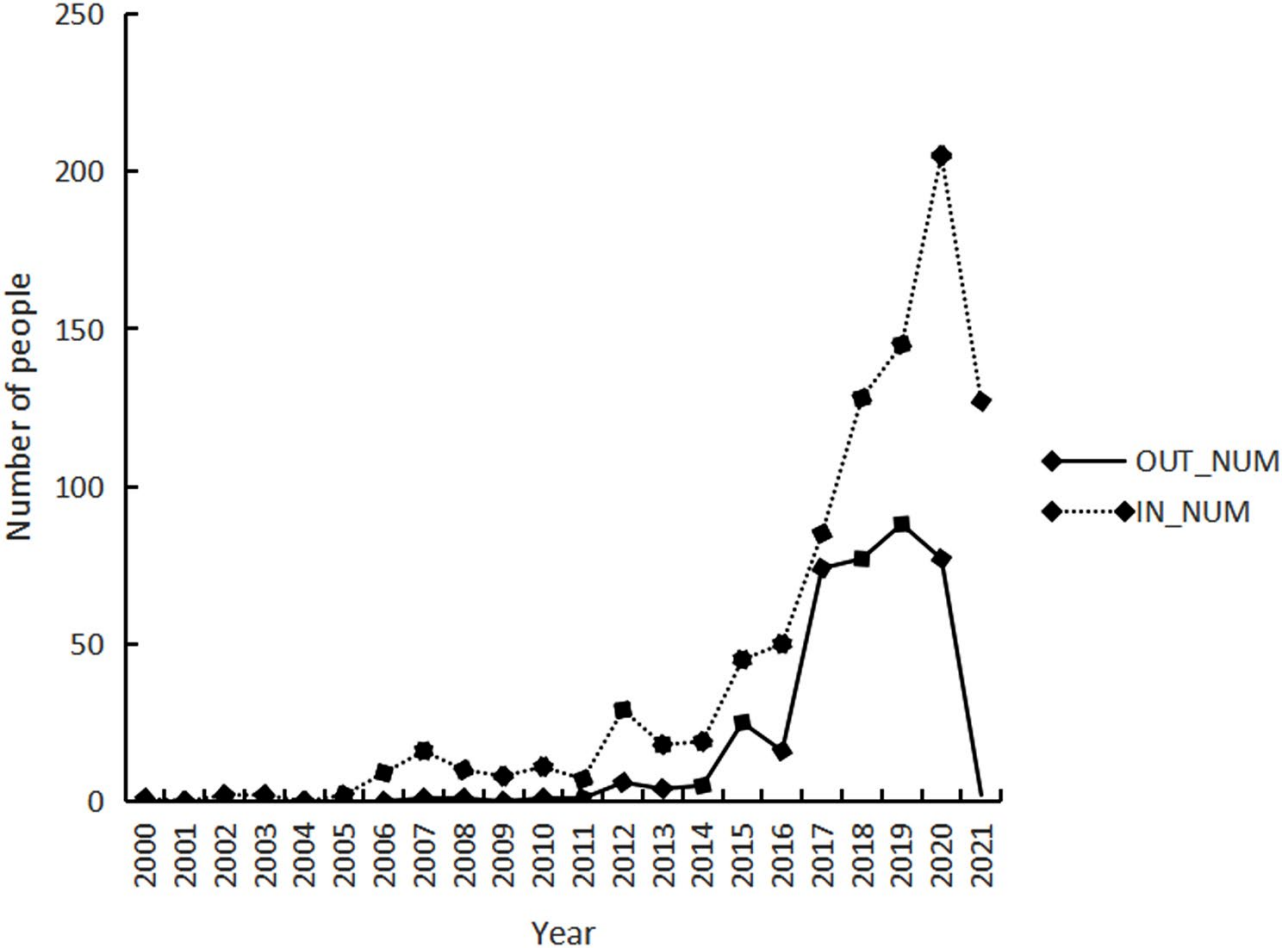
CHSS personnel turnover time distribution Chart

Between 2000 and 2021, there were increasing personnel in CHSSs. From 2000 to 2008, the stations primarily witnessed an influx of personnel, with a noticeable peak in 2007. This trend persisted from 2009 to 2011. Despite this, between 2012 and 2015, personnel mobility accelerated, in which both inflows and outflows rose dramatically. The year 2014 marked a turning point in this movement. By 2015, there was a sharp surge in both incoming and outgoing personnel. Later, the period from 2016 to 2020 saw the highest mobility among all the phases, with the largest influx recorded in 2020, welcoming 205 personnel. Conversely, 2019 registered the highest outflow with 88 departures. In 2021, the outflow of personnel sharply dropped, while inflows moderately declined. A comprehensive visual representation is available in Fig. 2.

#### Community health service centers (CHSCs)

The total number of health personnel that entered the CHSC amounted to 2,479. Of these, 19.16% were male and 80.84% were female. Registered nurses represented 38.00% of the professionals while practicing physicians comprised 30.50%. It should be noted that 95.97% of these health professionals had not obtained the standardized training certificate for national resident physicians. For professional technical positions, 28.36% were at the associate level, 32.92% were at the teacher level, and 21.70% were at the intermediate level. In terms of educational background, 41.67% had completed specialized university courses or vocational schools, 38.32% held a bachelor’s degree, and 14.56% had finished secondary vocational training. In the meantime, a significant 86.69% were not registered in general medical professions, while 12.59% were. Merely 1.43% practiced in multiple locations. Remarkably, 47.44% of these health personnel joined the CHSC for diverse reasons, while 43.00% were transferred from other institutions.

On the contrary, the number of health personnel departing the CHSC was 1,145. The male and female ratios stood at 22.27% and 77.73%, respectively. Registered nurses represented 32.58%, while 31.62% were practicing physicians. A staggering 98.78% had not acquired the national resident physician standardized training certificate. Regarding professional technical ranks, 30.66% were associates, 26.11% were at the teacher level, and 22.10% were intermediates. Concerning education, 41.92% finished specialized university courses or vocational schools, 31.27% had bachelor’s degrees, and 20.87% completed secondary vocational training. Only 9.69% were registered in the general medical profession, with 89.96% not being registered. In addition, 60.26% did not practice at multiple locations. The reasons for leaving covered resignation or dismissal (44.02%), transfers to other health institutions (28.56%), and other reasons (17.64%).

**Fig. 3:**
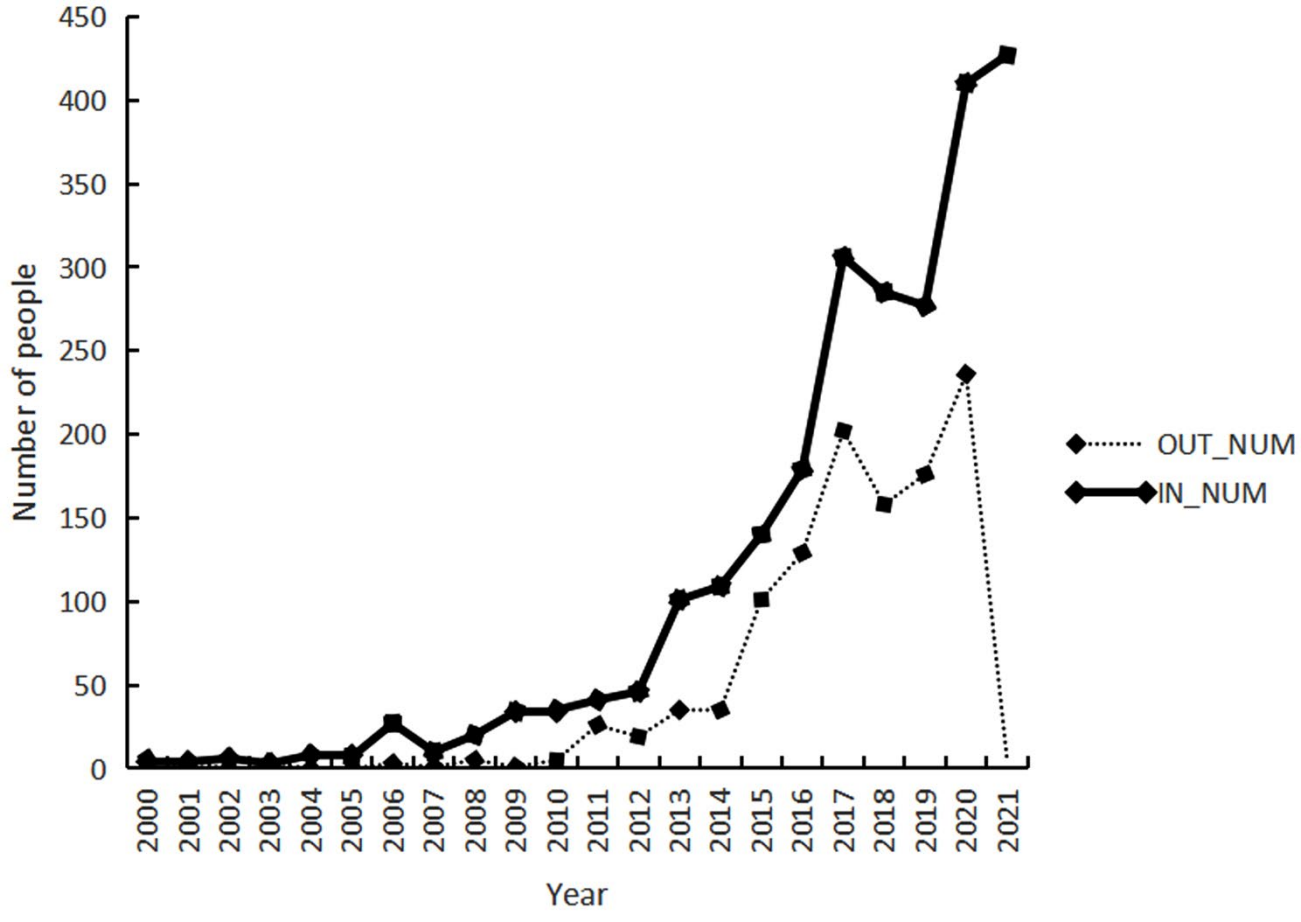
Temporal distribution of personnel mobility in CHSCs

From 2000 to 2008, there was minimal staff turnover in the CHSC. Nonetheless, the period between 2009 and 2011 marked a significant turning point, with a noticeable rise in personnel movement post this period. From 2012 to 2015, the flux of health personnel was considerably high, which represented a period of rapid growth. Between 2016 and 2020, the turnover peaked, with 2020 witnessing the highest influx, totalling 410, and simultaneously, the highest departure, totalling 236. Notably, in 2017, the personnel movement was relatively high, with 306 incoming and 202 outgoing staff, constituting a minor peak in that cycle as revealed in Fig. 3.

#### Township health centers (THCs)

A total of 9,580 healthcare professionals entered THCs, with men constituting 28.75% and women 71.25%. The professional categories of registered nurses, practicing physicians, and assistant physicians took up 32.47%, 13.41%, and 11.43% respectively. A dramatic 98.12% of these professionals did not possess the national residency standardization certification. Their professional technical positions at the junior, intermediate, and probationary levels comprised 33.12%, 23.60%, and 19.66% respectively. Regarding educational qualifications, those with junior college or vocational school, secondary vocational, and undergraduate degrees accounted for 47.81%, 28.11%, and 17.81% respectively. Notably, 90.80% were not registered in general medicine. Furthermore, 49.43% were not practitioners at multiple locations. The primary reasons for their influx into THCs were other reasons (64.07%) and graduation from higher or secondary institutions (20.07%).

Regarding the outflow, 1,180 healthcare professionals left the THCs. Men and women took up 30.13% and 69.87%, respectively. Additionally, registered nurses, practicing physicians, and other healthcare technical staff accounted for 27.48%, 14.62%, and 12.39% respectively. A vast 99.69% lacked the national residency standardization training certification. Their professional ranks at the junior, probationary, and intermediate levels were 33.27%, 29.40%, and 17.92% respectively. In addition, the highest educational qualifications observed were junior college or vocational school (39.25%), secondary vocational (39.53%), and undergraduate (11.98%). A significant 94.75% were not registered in general medicine, and 62.11% were not practitioners at multiple locations. The predominant reasons for their departure lie in resignations or dismissals (63.81%) and transfers to other healthcare institutions (20.50%).

**Fig. 4:**
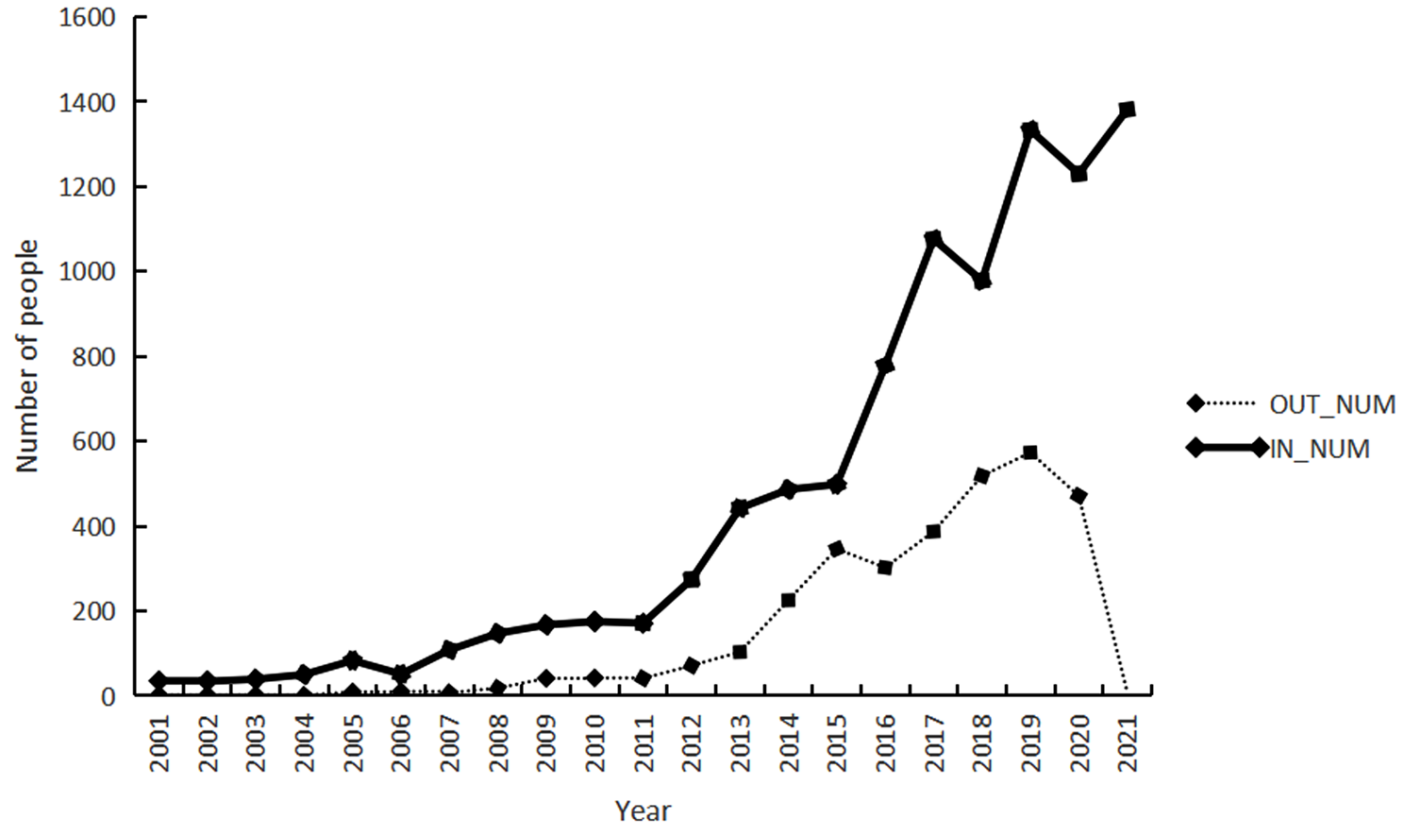
Temporal distribution of personnel mobility in THCs

Between 2000 and 2008, personnel mobility in THCs was relatively low. The year 2005 marked a significant turning point with noticeable rises in both influx and outflow post that year. After 2009, a consistent rise could be seen in both inflow and outflow in these centers. In 2021, the maximum inflow was observed with 1,381 individuals, whilst the outflow dramatically reduced to just nine individuals. In contrast, 2019 witnessed the highest outflow with 573 departures, as manifested in Fig. 4.

#### Village health centers

The total number of healthcare workers who moved into village health rooms is 1,260. Among them, 58.81% were males and 41.19% were females. 29.60% specialized in clinical practice. 43.10% did not possess a professional technical position, while 25.56% had a junior professional rank and 20.16% were on probation. 67.14% of them had an education level of junior college or vocational school, 18.41% had secondary vocational education, and 10.87% had undergraduate degrees. 20.32% of these healthcare workers were not registered in general medicine, while 73.57% were transferred from other institutions.

The number of healthcare workers who left the village health rooms totals 2,585. Among them, 67.89% were males and 32.11% were females. 51.33% specialized in clinical medicine and 39.69% in traditional Chinese medicine. 52.11% did not acquire a professional technical position, whereas 22.03% had a junior professional rank. 48.59% had an education level of junior college or vocational school, 26.48% had secondary vocational education, and 16.41% were not registered in general medicine. The primary reasons for healthcare workers leaving the village health rooms were retirement (33.98%), other reasons (26.41%), and resignation or dismissal (22.34%).

**Fig. 5:**
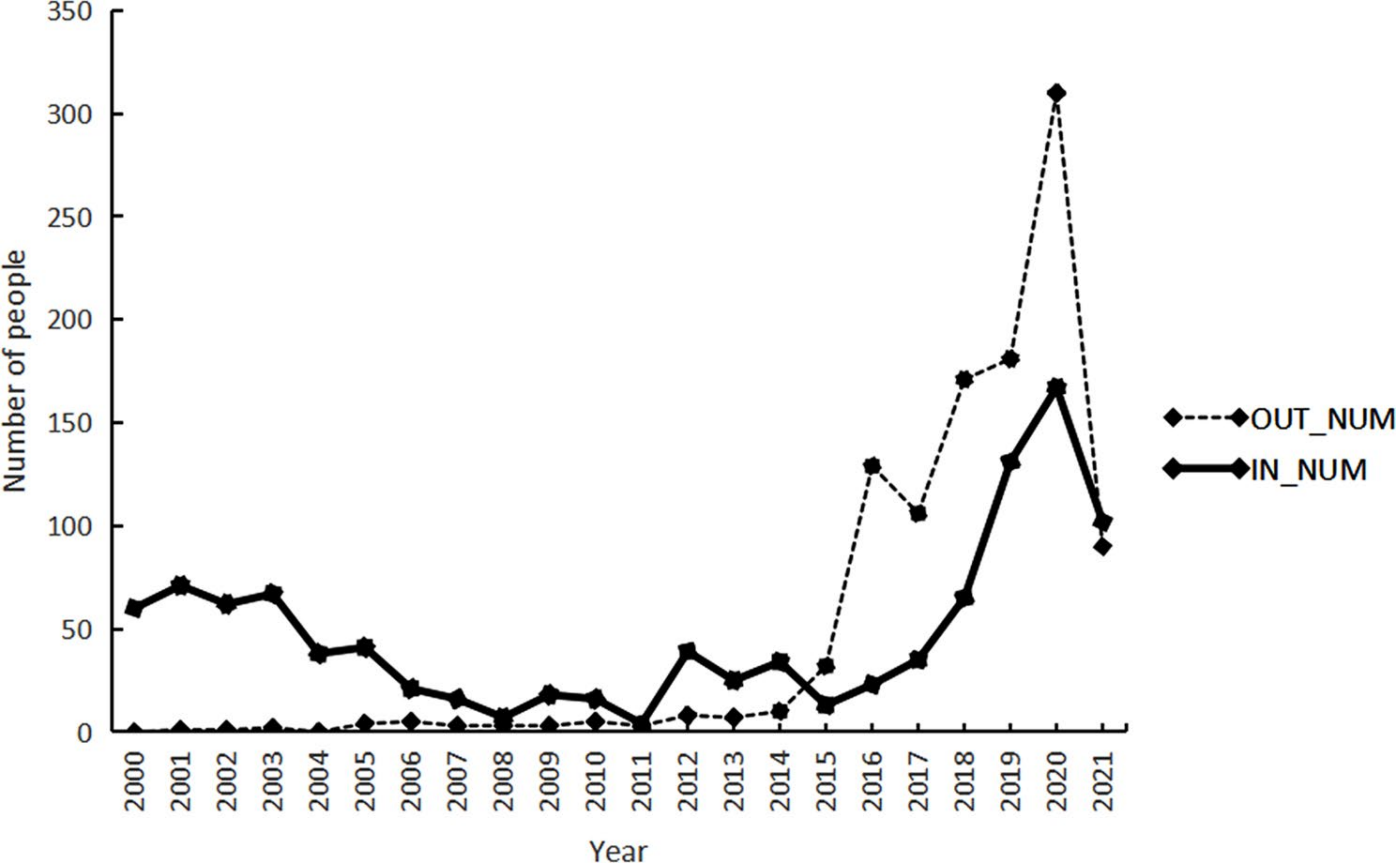
Temporal distribution of personnel mobility in village health center

Figure 5 shows that overall, the flow of healthcare workers in village health rooms differs from other primary health institutions. Before 2014, the influx of healthcare workers to village health rooms was more evident than the outflow. Nevertheless, after 2014, the outflow surpassed the influx. During 2000 and 2008, the influx displayed a declining trend, while the outflow remained relatively unchanged. During 2009 and 2014, the movement of healthcare workers in village health rooms was at a lower level. After 2014, the number of healthcare workers leaving village health rooms exceeded those coming in. Specifically, in 2020, the village health rooms experienced the highest movement with 310 healthcare workers leaving and 167 joining. Later in 2021, the inflow of healthcare workers in village health rooms once again surpassed the outflow.

### Characteristics of institutional types affecting the mobility of primary healthcare personnel

#### Frequency characteristics of movement to other institutions

**Fig. 6:**
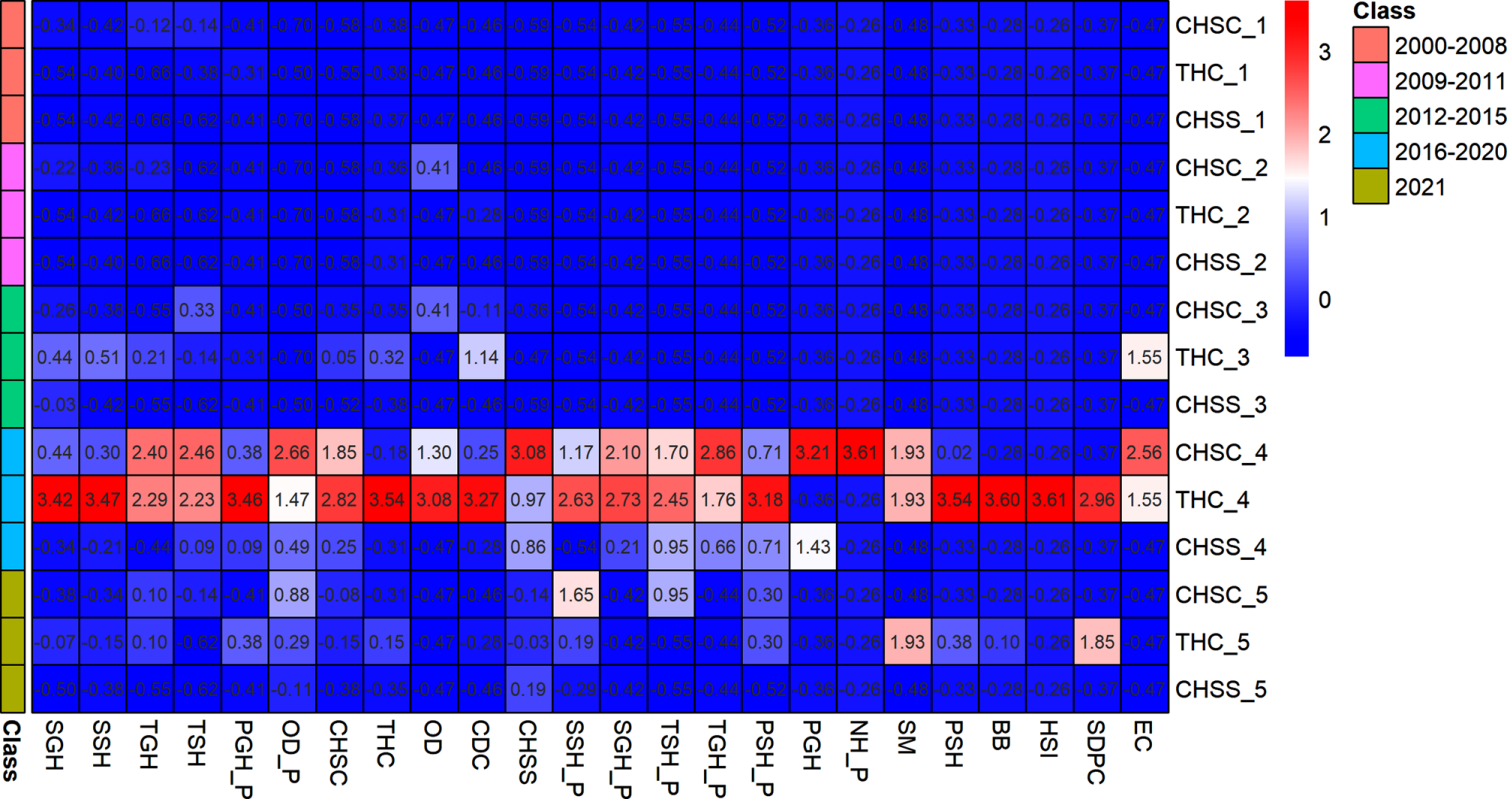
Characteristics of staff movement frequency among different healthcare institutions *Note* the names of different institutions are abbreviated

Figure 6 indicates that during the period from 2016 to 2020, the frequency of staff mobility among primary healthcare workers was at its highest, with the most significant movement occurring between THCs and other institutions, subsequently followed by CHSCs. In this timeframe, the exchange of personnel between THCs alone reached 477 instances. Movement of primary healthcare workers from these centers to secondary general hospitals happened 101 times, and to secondary specialty hospitals, 184 times, while transfers amongst CHSCs were recorded 73 times.

#### Characteristics of personnel movement to other institutional types

**Fig. 7:**
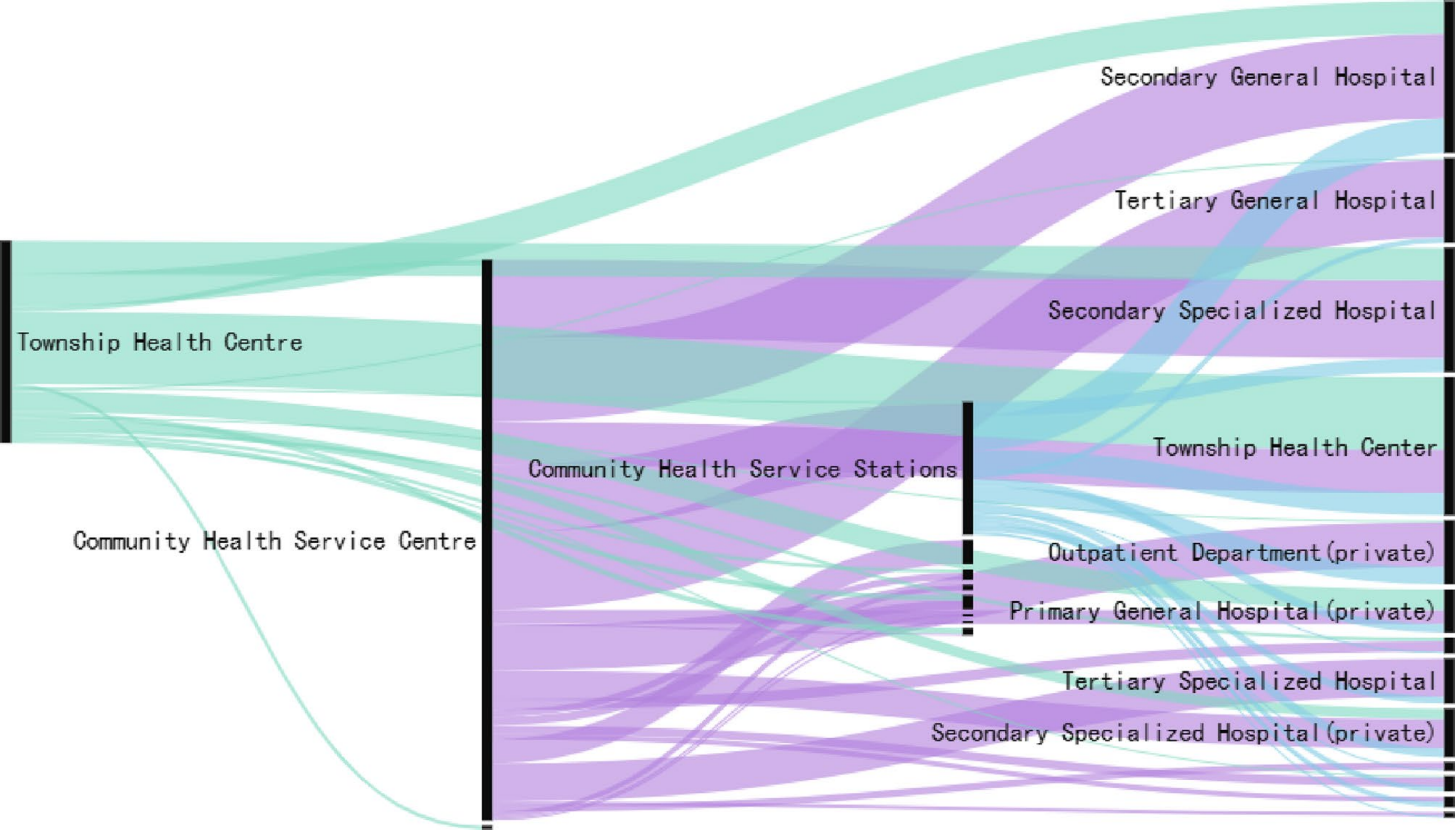
Sankey diagram illustrating the institutional transitions of primary healthcare personnel

Figure 7 indicates that the primary movement of grassroots health personnel, aside from their transition to primary medical institutions, predominantly directs towards secondary general hospitals, tertiary general hospitals, secondary specialized hospitals, private outpatient departments, private primary hospitals, tertiary specialized hospitals, and private secondary specialized hospitals.

#### Characteristics of primary healthcare workers transitioning to non-primary medical institutions

In the data presented in Table 1, altogether 925 individuals transitioned to non-primary medical institutions. Of these, 22.28% were male and 77.62% were female. From the perspective of ethnicity, Zhuang and Han nationalities constituted 40.11% and 58.05% respectively. In terms of years of service, those with experience less than five years took up 6.38%, those with 5–10 years made up 36.86%, and those with over ten years represented 56.76%. Professionally, registered nurses comprised 40.43%, while licensed doctors accounted for 25.08%. Regarding professional technical positions, 36.54% held junior professional titles, 29.30% held intermediate titles, 18.38% were awaiting appointments, and 12.65% held mid-level professional titles. The highest educational level attained was junior college for 44.32%, secondary vocational or technical school for 29.62%, and undergraduate degree for 24.54%. Geographically, 13.08% of the primary medical personnel were in Binyang County, 12.22% in Wuming District, and 11.14% in Hengzhou City. A vast majority of 99.14% had no national qualification certificate from standardized residency training, and 94.05% were not registered in general medical practice. Additionally, 22.38% of health personnel moved to secondary general hospitals, whilst 31.24% transitioned to secondary specialized hospitals.

**Table 1:** Basic Characteristics of mobile personnel (n, %)

	Total (*n*_1_ = 1954)	Non-primary health institutions (*n*_2_ = 925)
**Gender**		
Male	542(27.74)	207(22.38)
Female	1412(72.26)	718(77.62)
**Ethnicity**		
Zhuang	1169(59.83)	537(58.05)
Han	743(38.02)	371(40.11)
Other	42(1.94)	17(1.73)
**Years of work experience**		
Less than 5 years	126(6.45)	59(6.38)
5–10 years	784(40.12)	341(36.86)
More than 10 years	1044(53.43)	525(56.76)
**Professional category**		
Registered nurses	676(34.60)	374(40.43)
Practicing physicians	439(22.47)	232(25.08)
Other health technical staff	202(10.34)	71(7.68)
Assistant practicing physicians	187(9.57)	63(6.81)
Laboratory technicians (Junior Prof.)	77(3.94)	42(4.54)
Pharmacists in western medicine (Junior Prof.)	65(3.33)	37(4.00)
Trainee physicians	138(7.06)	36(3.89)
Other technical personnel	55(2.81)	21(2.27)
Radiology technicians (Junior Prof.)	48(2.46)	18(1.95)
Management personnel	19(0.97)	12(1.30)
Skilled labor personnel	28(1.43)	12(1.30)
Midwives	13(0.67)	4(0.43)
TCM pharmacists (Junior Prof.)	7(0.36)	3(0.32)
**Professional technical position code**		
None	31(1.59)	14(1.51)
Junior level	731(37.41)	338(36.54)
Intermediate/senior assistant	503(25.74)	271(29.30)
Pending appointment	449(22.98)	170(18.38)
Intermediate level	212(10.85)	117(12.65)
Sub-senior level	27(1.38)	15(1.62)
Senior level	1(0.05)	0(0.00)
**Highest educational attainment**		
Junior college	883(45.19)	410(44.32)
Secondary specialized and technical education	663(33.93)	274(29.62)
Bachelor’s degree	384(19.65)	227(24.54)
Graduate degree	19(0.97)	13(1.41)
High school	4(0.20)	1(0.11)
Middle school and below	1(0.05)	0(0.00)
**Administrative division code**		
Binyang county	202(10.34)	121(13.08)
Wuming district	237(12.13)	113(12.22)
Hengzhou city	227(11.62)	103(11.14)
Shanglin county	154(7.88)	94(10.16)
Qingxiu district	180(9.21)	90(9.73)
Xixiangtang district	210(10.75)	84(9.08)
Jiangnan district	162(8.29)	75(8.11)
Liangqing district	139(7.11)	69(7.46)
Long’an county	153(7.83)	66(7.14)
Mashan county	121(6.19)	65(7.03)
Xingning district	99(5.07)	27(2.92)
Yongning district	70(3.58)	18(1.95)
**National standardized resident doctor training certification**		
Yes	17(0.87)	8(0.86)
No	1937(99.13)	917(99.14)
**Registered in general medical practice**		
Yes	165(8.44)	54(5.84)
No	1778(90.99)	870(94.05)
Not applicable	11(0.56)	1(0.11)
**Type of inflowing institution**		
Secondary general hospital	207(10.59)	207(22.38)
Secondary specialized hospital	289(14.79)	289(31.24)
Tertiary general hospital	90(4.61)	90(9.73)
Primary general hospital (Privately-owned)	62(3.17)	62(6.70)
Disease prevention and control center	39(2.00)	37(4.00)
Other Non-primary health care institutions	536(27.43)	240(25.95)
Township health center	388(19.86)	-
Community health service center	264(13.51)	-
Community health service station	79(4.04)	-

#### Determinants influencing the transition of primary healthcare personnel to non-primary healthcare institutions

As presented in Table 2, after discarding records with missing values, a final sample size of 1912 was attained. The logistic regression outcomes reveal that primary healthcare workers who are male have a 66.5% likelihood of transitioning to non-primary healthcare institutions compared to females. For the highest level of educational attainment, the likelihood of health personnel possessing graduate degrees, undergraduate degrees, and associate degrees moving to non-primary healthcare facilities are 5.422, 3.316, and 1.674 times greater, respectively, than those with secondary vocational and technical education. Primary healthcare workers of Han ethnicity are 1.255 times more likely to move to non-primary healthcare institutions than those of other ethnic groups. Moreover, those in the role of intern physicians have a 25.3% likelihood of transitioning compared with administrative staff, and those registered as general medical practitioners have a 2.922 times greater likelihood of moving to non-primary healthcare institutions than those who are not. Additionally, regional variance can be observed in the propensities of primary healthcare workers to shift to non-primary healthcare settings. In comparison to Hengzhou City, staff from the three urban districts of Xingning, Xixiangtang, and Yongning are less likely to move to non-primary healthcare institutions, whereas those from the three counties of Mashan, Binyang, and Shanglin are more inclined to such transitions.

**Table 2:** Factors influencing the mobility of primary healthcare workers to non-primary healthcare institutions

	B	Sig.	Exp (B)	95% C.I. for EXP(B)	
				Lower	Upper
Gender (ref: female)	-0.407	0.002	0.665	0.514	0.862
Highest Education (ref: Secondary Technical)					
Graduate Degree	1.690	0.002	5.422	1.850	15.891
Bachelor’s Degree	1.199	0.000	3.316	2.357	4.666
Associate Degree	0.515	0.000	1.674	1.318	2.125
Ethnicity (ref: other)					
Han	0.227	0.048	1.255	1.002	1.572
Zhuang	0.108	0.791	1.114	0.501	2.478
Years of Service (ref: >10 years)					
< 5 years	0.127	0.564	1.136	0.737	1.750
5–10 years	-0.101	0.389	0.904	0.717	1.138
Professional Category (ref: Administrative Staff)					
Practicing Physician	-0.274	0.614	0.760	0.261	2.211
Practicing Assistant Physician	-0.993	0.075	0.371	0.124	1.105
Intern Physician	-1.374	0.017	0.253	0.082	0.782
Registered Nurse	-0.157	0.772	0.854	0.295	2.477
Midwife	-1.126	0.173	0.324	0.064	1.637
Western Pharmacist (Assistant)	-0.158	0.790	0.854	0.267	2.732
Chinese Medicine Pharmacist (Assistant)	-0.509	0.602	0.601	0.089	4.059
Laboratory Technician (Assistant)	-0.151	0.797	0.859	0.272	2.720
Radiology Technician (Assistant)	-0.878	0.154	0.416	0.124	1.391
Other Health Technicians	-0.886	0.116	0.412	0.137	1.243
Other Technicians	-0.913	0.135	0.401	0.121	1.329
Professional Title (ref: Pending Appointment)					
Associate Senior	0.347	0.454	1.414	0.571	3.502
Intermediate	0.385	0.098	1.470	0.932	2.319
Primary/Assistant	0.288	0.120	1.333	0.928	1.915
Junior	0.113	0.450	1.119	0.835	1.500
National Residency Training Certificate	0.324	0.544	1.383	0.486	3.934
Registered in General Practice	1.072	0.000	2.922	1.937	4.408
Administrative Division (ref: Hengzhou City)					
Xingning District	-1.128	0.000	0.324	0.185	0.566
Qingxiu District	-0.281	0.201	0.755	0.491	1.162
Jiangnan District	-0.430	0.059	0.651	0.417	1.016
Xixiangtang District	-0.642	0.003	0.526	0.344	0.805
Liangqing District	-0.310	0.195	0.734	0.459	1.172
Yongning District	-1.377	0.000	0.252	0.129	0.492
Wuming District	-0.042	0.840	0.959	0.637	1.444
Longan County	0.092	0.693	1.097	0.694	1.734
Mashan County	0.524	0.034	1.688	1.040	2.742
Shanglin County	0.527	0.025	1.695	1.068	2.689
Binyang County	0.644	0.003	1.904	1.253	2.895
Constant	-1.429	0.073	0.240		

All the significance levels were set at a *P*-value < 0.05

### Analysis of geographic characteristics of primary health personnel mobility

#### Geographic characteristics of influx of primary health personnel

**Fig. 8:**
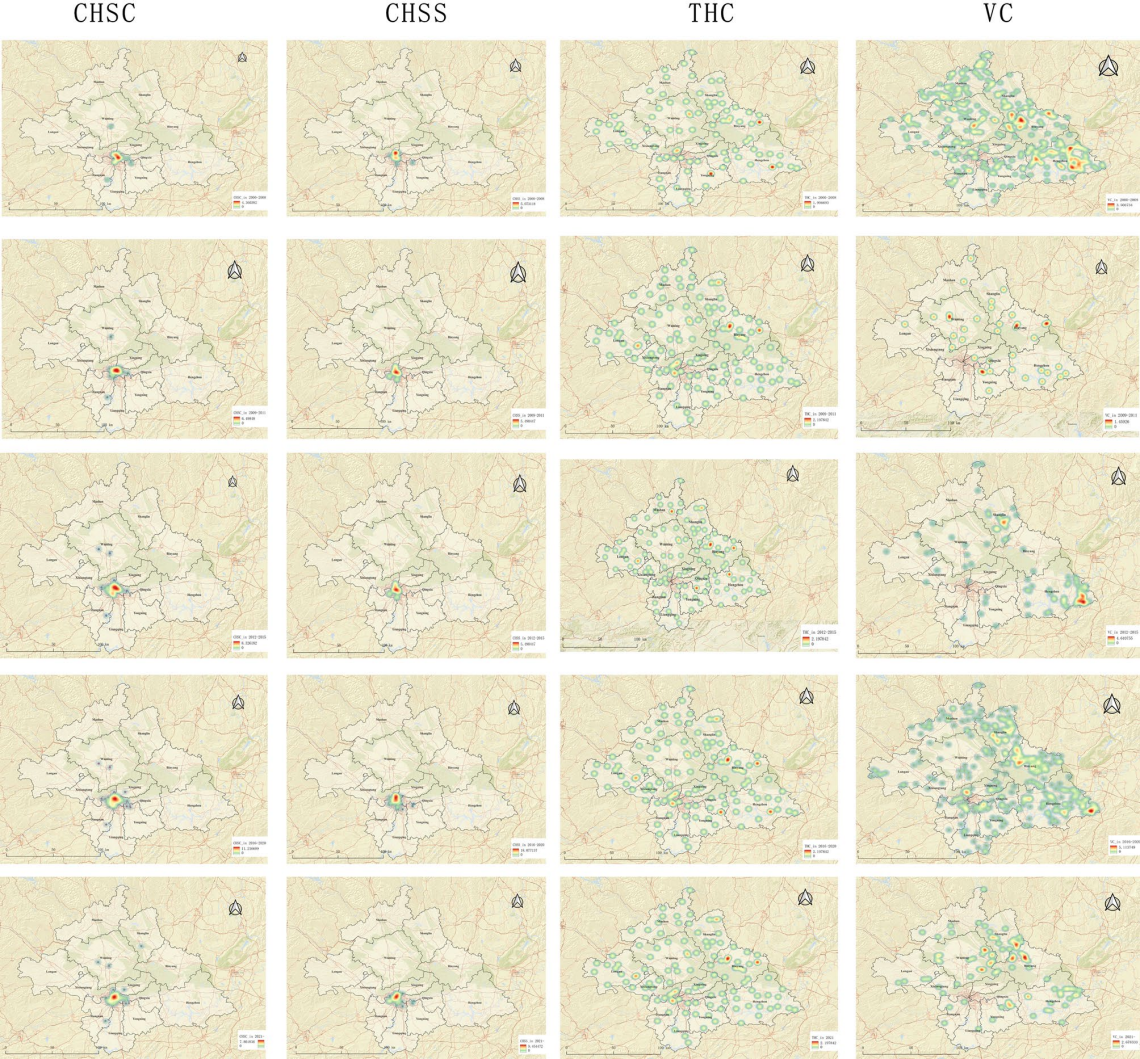
Heat map of inflow points for primary health personnel. *Note* this figure presents point heat maps for five distinct stages, namely 2000–2008, 2009–2011, 2012–2015, 2016–2020, and 2021, arrayed in rows from 1 to 5

As depicted in Fig. 8, the influx of personnel to CHSCs is primarily concentrated at the confluence of Qingxiu District, Xingning District, Xixiangtang District, Jiangnan District, Liangqing District, and Yongning District. This region boasts a substantial concentration of medical personnel in its healthcare institutions, radiating out to several nodal core areas in the periphery. What began as scattered nodal cores in Wuming District and Jiangnan District in the initial phase has expanded by the fifth phase to involve multiple fragmented nodal cores in areas, such as Wuming District, Jiangnan District, Shanglin County, and Xingning District. By the same token, the inflow of personnel to CHSSs mainly clusters around the borders of several districts, with barely any change in the thermal core areas across the five stages. The inflow of personnel to THCs does not exhibit a particular concentration in any area, and the characteristics of personnel inflow to village health posts are similar.

#### Geographic characteristics of outflow of primary health personnel

**Fig. 9:**
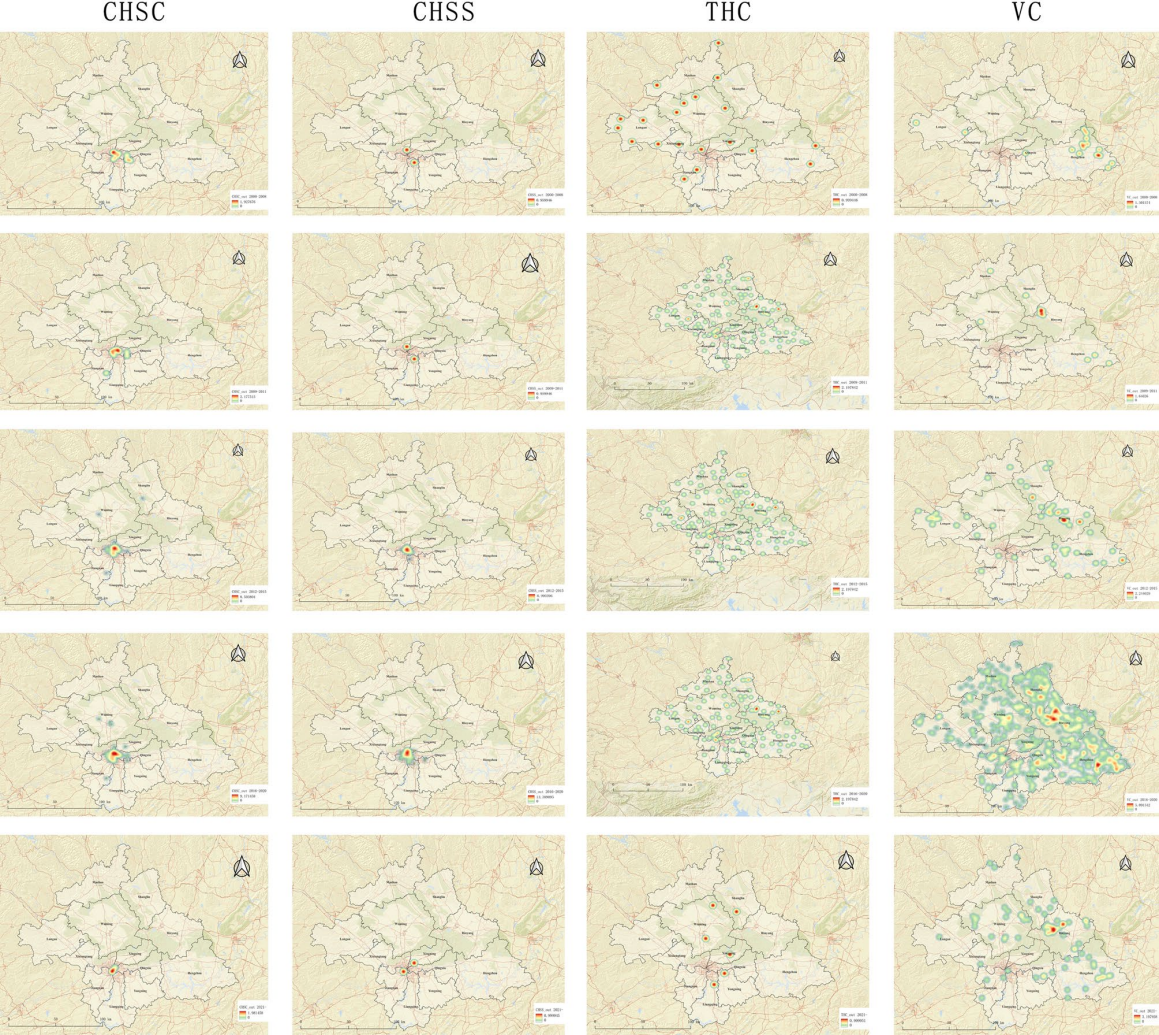
Heat Map of pnflow points for primary health personnel. *Note* this figure presents point heat maps for five distinct stages, namely 2000–2008, 2009–2011, 2012–2015, 2016–2020, and 2021, arrayed in rows from 1 to 5

Figure 9 presents that the exodus centers of primary health personnel from CHSCs are predominantly concentrated at the intersections of Qingxiu District, Xingning District, Xixiangtang District, Jiangnan District, Liangqing District, and Yongning District. The outflow hubs of personnel from CHSSs were mainly clustered towards the intersections of the six districts, particularly around Xixiangtang and Liangqing Districts during the years 2000–2008 and 2009–2011. During the 2012–2015 and 2016–2020 periods, the exodus centers were primarily concentrated at the junctions of the six districts. In 2021, there were two main outflow centers, including one in Xingning District and the other leaning towards the intersection of the six districts in Jiangnan District. Furthermore, the personnel outflow from THCs and VCs was relatively dispersed.

#### Analysis of the shift in the gravitational center of primary healthcare personnel

**Fig. 10:**
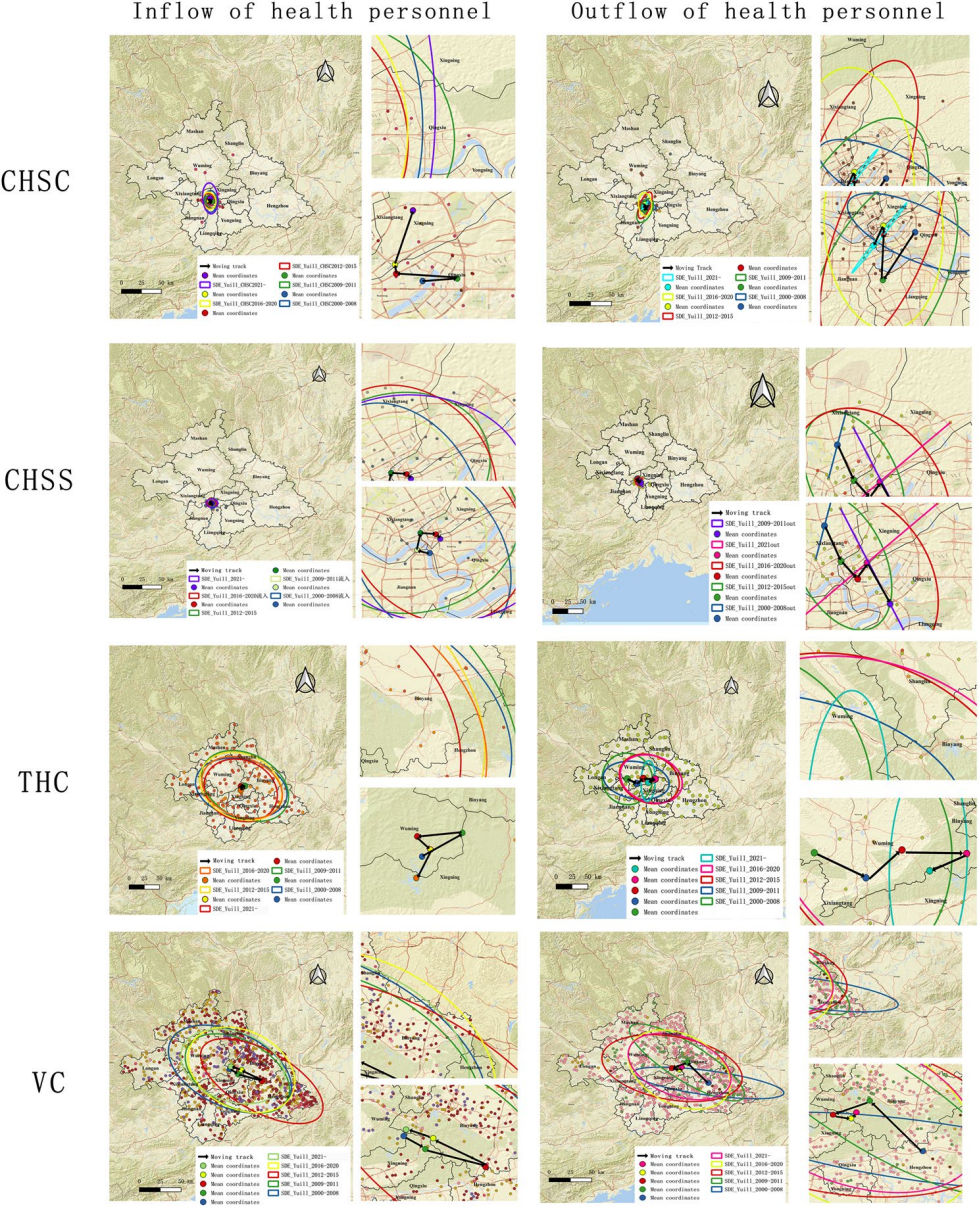
Primary healthcare personnel migration center of gravity map

As shown in Fig. 10, the center of inflow for personnel in CHSCs has shifted predominantly northward. The outflow center for these centers initially moved to the southwest after the initial stage, followed by a general shift northward from the second to the fourth stages, and a slight southward adjustment in the fifth stage. The inflow center for CHSSs has moved clockwise to the northeast, while the overall outflow center has shifted to the southeast. For THCs, the center of health personnel inflow moved to the northeast after the first stage, followed by shifting westward after the second stage, and then generally southward after the third stage. Specifically, the overall outflow of health personnel from these centers moved eastward, in which a southwestward shift followed the fourth stage. The inflow center for village health rooms initially moved southeastward, with a transition to the northwest after the third stage. In general, the outflow center for health personnel generally moved toward the northwest, which transitioned to the southwest after the first stage, then to the southeast after the third, and northeast after the fourth stage.

#### Spatial autocorrelation analysis of primary healthcare personnel

As can be observed in Table 3, Moran’s I indices for the inflow and outflow of healthcare personnel from the four primary healthcare institutions are not significant, which suggests no spatial clustering in the movement of primary healthcare personnel.

**Table 3:** Analysis of spatial clustering in the mobility of primary healthcare institution personnel

		2016	2017	2018	2019	2020
CHSC_in	Morans’ I	-0.307	-0.103	-0.132	-0.239	-0.171
	SD	0.093	0.088	0.094	0.095	0.089
	P	0.677	0.416	0.455	0.592	0.506
CHSC_out	Morans’ I	-0.370	-0.248	-0.174	-0.186	0.007
	SD	0.099	0.096	0.087	0.092	0.092
	P	0.740	0.603	0.510	0.526	0.283
CHSS_in	Morans’ I	-0.412	-0.262	0.171	0.173	0.157
	SD	0.081	0.087	0.073	0.093	0.089
	P	0.806	0.626	0.106	0.132	0.138
CHSS_out	Morans’ I	-0.307	-0.103	-0.132	-0.239	-0.171
	SD	0.093	0.089	0.094	0.095	0.089
	P	0.677	0.416	0.455	0.592	0.506
THC_in	Morans’ I	-0.215	0.067	0.057	0.049	-0.026
	SD	0.027	0.029	0.024	0.030	0.038
	P	0.774	0.176	0.172	0.230	0.369
THC_out	Morans’ I	0.215	0.164	0.082	0.121	-0.037
	SD	0.046	0.041	0.046	0.043	0.046
	P	0.077	0.105	0.209	0.153	0.401
VC_in	Morans’ I	0.002	-0.079	-0.063	-0.020	-0.071
	SD	0.031	0.044	0.046	0.031	0.014
	P	0.298	0.477	0.449	0.344	0.432
VC_out	Morans’ I	-0.063	-0.068	0.086	-0.040	-0.020
	SD	0.038	0.041	0.044	0.044	0.037
	P	0.443	0.456	0.199	0.404	0.357

#### Trajectory analysis of primary healthcare personnel mobility

**Fig. 11:**
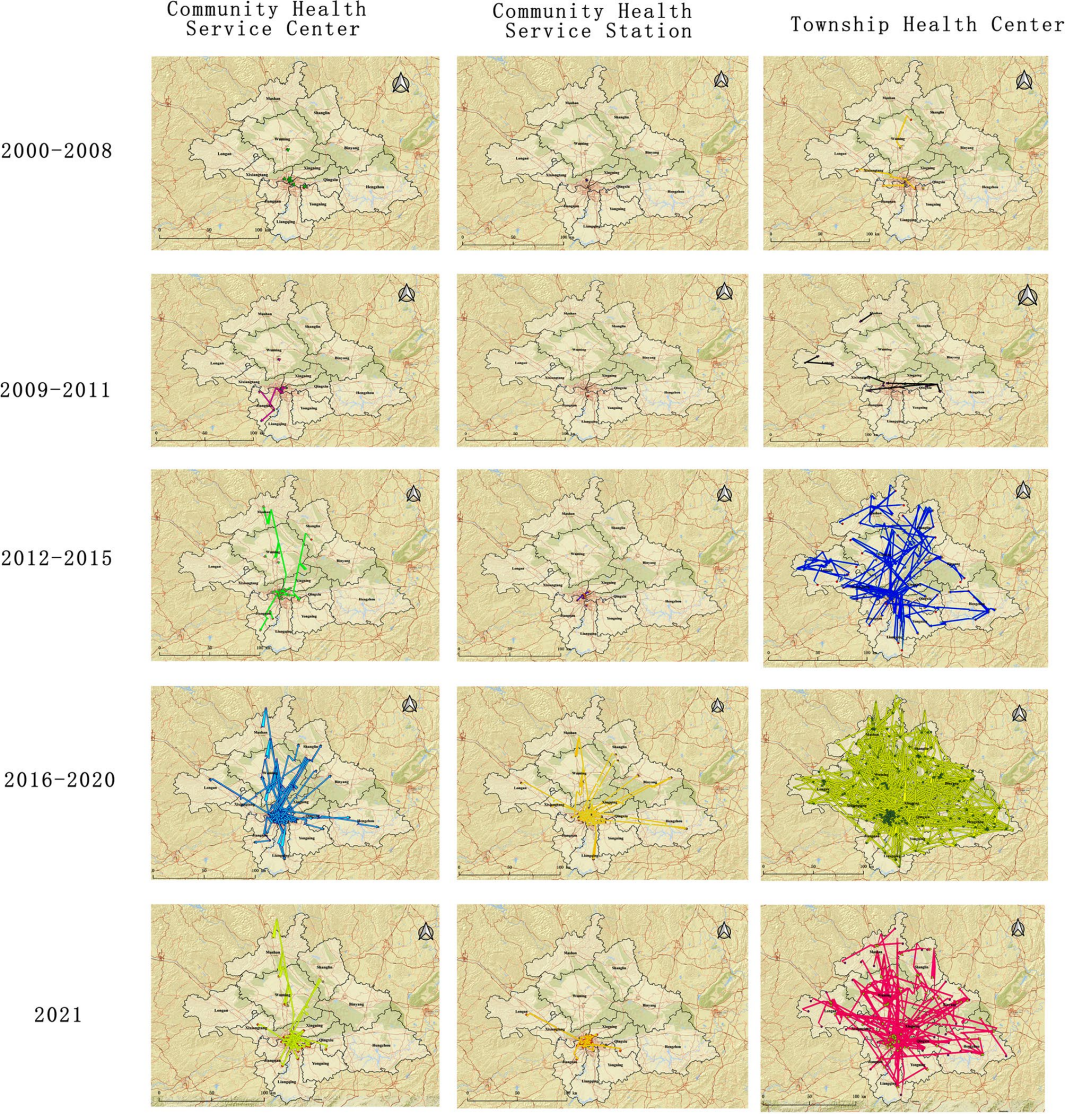
Primary healthcare personnel migration map

Figure 11 reveals that the number and extent of migration among primary healthcare personnel at grassroots medical and health institutions were relatively minor during 2000 and 2011. The staff migration at CHSSs remained modest in number and scale during 2012 and 2015. On the contrary, the migration magnitude for personnel at CHSSs and THCs became noticeably larger. From 2016 to 2021, the migration magnitude for healthcare personnel in primary medical and health institutions remained at a higher level. Table 4 implies that the main migration of primary healthcare personnel occurred in their own district (county).

**Table 4:** Top 10 destinations of primary healthcare personnel migration

Rank	CHSCs	CHSSs	THCs
1	Qingxiu District - Qingxiu District	Xixiangtang District - Xixiangtang District	Hengzhou City - Hengzhou City
2	Jiangnan District - Jiangnan District	Liangqing District - Liangqing District	Wuming District - Wuming District
3	Wuming District - Wuming District	Xixiangtang District - Xingning District	Binyang County - Binyang County
4	Xixiangtang District - Xixiangtang District	Xingning District - Xingning District	Shanglin County - Shanglin County
5	Qingxiu District - Xingning District	Qingxiu District - Qingxiu District	Long’an County - Long’an County
6	Liangqing District - Liangqing District	Xixiangtang District - Jiangnan District	Mashan County - Mashan County
7	Jiangnan District - Liangqing District	Jiangnan District - Xixiangtang District	Xixiangtang District - Xixiangtang District
8	Jiangnan District - Xixiangtang District	Liangqing District - Yongning District	Yongning District - Yongning District
9	Qingxiu District - Xixiangtang District	Qingxiu District - Liangqing District	Liangqing District - Liangqing District
10	Jiangnan District - Qingxiu District	Xixiangtang District - Qingxiu District	Xingning District - Xingning District

## Discussion

In this research, it was observed that the mobility of primary health personnel at CHSCs and township health clinics in Nanning from 2000 to 2021 can be categorized into four distinct phases: an initial stage (2000–2008), a turning point (2009–2011), a rapid development stage (2012–2020), and a brief decline (2021). The mobility of primary health personnel in VCs and CHSSs was at its initial stage from 2000 to 2011, which experienced a turning point from 2012 to 2015 and a rapid development stage from 2016 to 2021. During the initial stage, the features of personnel mobility were marked by a limited number of movements and short distances. The turning point was characterized by the rise of personnel movements and an expansion in the distances traveled. The rapid development stage was distinguished by the highest levels of mobility regarding both the number of personnel moving and the distances they traveled.

The shift in the mobility trend for other medical personnel during 2009 and 2011 can be attributed to the new healthcare reform initiated in 2009, which was intended to decentralize general medical services to the primary level, and gradually implement community-based first consultations, tiered medical treatment and two-way referrals. Despite this, owing to financial constraints, primary healthcare institutions could not acquire advanced medical equipment, resulting in difficulties in diagnosing certain conditions. Additionally, these institutions were required to comply with the national essential drug list, which caused the unavailability of many medications at the primary level, necessitating patients to seek treatment at higher-level hospitals. This gave rise to a dramatic drop in patient trust in primary healthcare facilities, a drop in outpatient visits, and then a substantial outflow of primary healthcare personnel [12]. When seeking employment, medical students consider such factors as salary, prospects for development, and surrounding amenities. Most medical students do not opt for positions in primary healthcare institutions [19], Instead, they prefer to work in urban tertiary hospitals, with many viewing employment at primary healthcare facilities merely as a transitional role. These reasons have caused an increase in the turnover of primary healthcare personnel after 2009.

In 2021, the COVID-19 pandemic resulted in fewer external job opportunities, and accordingly, the outflow of staff from primary healthcare institutions declined. VCs in Nanning began implementing the “Township Recruitment, Village Employment” policy in the second half of 2020. This policy reformed the income structure of rural doctors, adopting a system of basic salary plus performance wages and providing social insurance contributions for rural doctors, which facilitated their transition from part-time farmers and part-time medical practitioners to professional rural physicians. The integrated rural policy reduced the inflow of rural doctors and lifted their retention, reversing the trend of more doctors leaving than entering rural practice. This policy is beneficial for enhancing health equity in rural areas.

Finishing the new healthcare reform to encourage more primary healthcare personnel to work in grassroots medical institutions, China has successively introduced many policies from the perspectives of medical education, salary, and professional title promotion, but there were no significant effects. In the aspect of medical education, China implemented the Rural-oriented Tuition-waived Medical Education policy in 2010. Students under this policy are required to complete five years of medical education, followed by three years of standardized training, and then serve for three years at township health clinics. As identified in existing research, a significant number of these medical students report low job satisfaction [20, 21] with a pronounced intention to resign [22]. The principal reasons cited for this discontent are the low levels of pay, long working hours, scarce opportunities for further training, and limited prospects for career advancement. In terms of salary, before 2015, primary healthcare institutions operated under a “separate revenue and expenditure” policy, There was a very low proportion of healthcare workers’ performance pay compared to their basic salary, lacking incentives, which diminished their enthusiasm for offering clinical services and even resulted in the exodus of core clinical staff from these grassroots institutions. After 2015, this policy was gradually abolished, while its effects require time to dissipate. In 2017, the Chinese government’s “Two Permissions” policy aimed to provide positive guidance on the remuneration of primary healthcare institutions. Despite this, a great number of grassroots healthcare facilities have struggled to effectively implement this due to a lack of concrete and actionable measures. Numerous studies have demonstrated that satisfaction with compensation and job satisfaction are significant factors that affect the professional loyalty of primary healthcare workers [23, 24]. China’s healthcare system remains predominantly centered around large hospitals [25, 26] with primary healthcare institutions receiving comparatively less emphasis. Hence, the salaries offered by primary healthcare institutions are often insufficient to satisfy primary healthcare personnel, who may opt for non-primary healthcare institutions in pursuit of higher wages. In terms of professional promotion, the proportion of mid-to-high-level professional titles among primary healthcare personnel is relatively low, which makes career breakthroughs difficult. This study utilizes data on the mobility of primary healthcare personnel to examine the influencing factors of the transition of primary healthcare workers to non-primary healthcare institutions. The research identifies the principal determinants for primary healthcare personnel moving to non-primary healthcare settings, which cover gender, educational level, ethnicity, professional category, registration as a general practice professional, and their location (district or county). Except for VHCs where the number of male personnel moving exceeds that of females, in other primary healthcare institutions, the mobility of female staff goes beyond that of males. Primary healthcare workers from county areas have a higher likelihood of transitioning to non-primary healthcare institutions, whereas those from urban districts are less inclined to make such moves. Previous research suggests that younger primary healthcare personnel may migrate to higher-level hospitals for the sake of their offspring’s education, despite potentially higher salaries at THCs, owing to the generally lower standard of education available in rural areas compared to urban centers [10]. This study reveals that the predominant movement of primary healthcare workers occurs in their county, rather than being drawn to urban hospitals in large numbers [27]. Most of these healthcare workers migrate to other THCs in the same locality, with the secondary hospitals being the main non-primary healthcare institutions they move to. Given that most personnel in THCs hold educational qualifications of a bachelor’s degree or less, these primary healthcare workers typically do not leave the primary healthcare sector.

Healthcare personnel with higher qualifications have a higher likelihood of being drawn away by superior medical institutions given the broader array of choices available to them. Consequently, the government should not only expand recruitment efforts aimed at healthcare workers with qualifications below the undergraduate level but also bolster the development of such personnel, who are poised to become the backbone of primary healthcare services. For those with undergraduate degrees and higher, it is vital to create additional opportunities for professional advancement, which can solidify the ranks of primary healthcare workers and enhance the quality of services provided to the grassroots population.

In Nanning, a city in a western minority region where ethnic minorities constitute 51.45% of the population, Han healthcare workers may be confronted with difficulties assimilating due to language barriers and cultural disparities. Many elderly members of the Zhuang minority in rural areas have a limited command of Mandarin, primarily communicating in their native Zhuang language, which poses challenges for Han healthcare workers in their interactions with patients, leading to attrition from primary healthcare institutions. In view of this, it is recommended that leaders of primary healthcare institutions intensify their humanitarian care for non-local healthcare workers and promptly address the living and working challenges faced by non-local Han healthcare staff.

There is minimal likelihood of intern physicians from primary healthcare institutions transitioning to non-primary medical facilities given the fact that interns are required to accomplish their practicum assignments as stipulated by their academic institutions to qualify for graduation, which reduces the probability of their migration to non-primary healthcare settings. Physicians registered in general practice medicine have a higher propensity to leave, which echoes the findings of Gan et al. [28], where the primary factors for general practitioners’ resignations cover low professional titles, inadequate remuneration, educational level, and night shifts. Although general practitioners in Nanning receive specific subsidies, the lack of transparency in the wage structure suggests that many are unaware that these subsidies are included in their salaries. Moreover, the variability of these subsidies across varying regions restricts their intended compensatory impact. Thus, it is recommended that the government should enhance the transparency of general practitioners’ subsidies and coordinate across all districts and counties of the city to establish clear and transparent rules for these subsidies, with preferential allocation directed towards remote areas and districts (counties) serving larger populations.

With an econo-geographic matrix to analyze the spatial correlation of primary healthcare staff mobility in Nanning from 2016 to 2020, this study revealed an absence of spatial clustering in the mobility of primary healthcare personnel in the city. Research manifests that post the healthcare reform of 2009 in China’s southwestern regions, a significant enhancement was witnessed in the equitable distribution of primary healthcare staff [29]. Despite increased governmental financial input and policies to attract talent, a poor working environment and limited promotion opportunities continue to impact the distribution equity of primary healthcare personnel in China’s southwest, with geographic equity still lacking. Equitable regional distribution of primary healthcare staff plays an important role in fairness, which in turn is conducive to strengthening patient satisfaction [30]. The government should persist in fostering the construction of primary healthcare talent pools in remote areas and regions with larger service populations to enhance the geographical equity of health services.

The limitation of this study lies in that owing to missing data regarding the year and institution in the personnel mobility database, it is impossible to describe the mobility characteristics of primary healthcare staff in Nanning with complete accuracy. The categories included in the primary healthcare staff are numerous, and due to incomplete data provided in the Nanning Statistical Yearbook, this paper only discusses the spatial clustering of primary healthcare staff from 2016 to 2020. The study did not explore the temporal and spatial mobility characteristics of primary healthcare physicians and nurses, nor did it examine whether there is spatial autocorrelation in their movement, whether personnel mobility in primary healthcare institutions in different regions exhibits spatial effects, or whether there is a spatial effect in the flow data of primary healthcare personnel. All these are aspects that should be addressed in future research on the temporal and spatial mobility characteristics and spatial effects of primary healthcare physicians and nurses.

Enhancing the development of the primary healthcare workforce remains a critical topic of research in the academic community in China [31–34]. This study provides evidence of primary healthcare personnel mobility during diverse stages of healthcare reform, which can assist policymakers in better understanding the demographic, geographic, and temporal characteristics of the movement of primary healthcare workers in Nanning over the past two decades, the principal institutions to which they migrate, and the major features of those who move to non-primary healthcare institutions. Meanwhile, the findings of this research can help both better evaluate past primary healthcare personnel policies and refine the focus of future primary healthcare human resource policies.

## Data Availability

The datasets utilized and/or analyzed in the present study can be obtained from the corresponding author upon reasonable request.

## Abbreviations

PHPD: The primary healthcare personnel database
DPAP: The demographic and professional attributes of the inflow and outflow personnel
SMPP: Specific migration patterns of the outgoing personnel
SGH: Secondary General Hospital
SSH: Secondary Specialized Hospital
TGH: Tertiary General Hospital
TSH: Tertiary Specialized Hospital
PGH_P: Primary General Hospital (private)
OD_P: Outpatient Department (private)
CHSC: Community Health Service Centre
THC: Township Health Center
OD: Outpatient Department
CDC: Centers for Disease Control and Prevention
CHSS: Community Health Service Station
SSH_P: Secondary Specialized Hospital (private)
SGH_P: Secondary General Hospital (private)
TSH_P: Tertiary Specialized Hospital (private)
TGH_P: Tertiary General Hospital (private)
PSH_P: Primary Specialized Hospital (private)
PGH: Primary General Hospital
NH_P: Nursing Home (private)
SM: Sanatorium
PSH: Primary Specialized Hospital
BB: Blood Banks
HSI: Health Supervision Institute
SDPC: Specialized Disease Prevention and Control Institute
EC: Emergency Centre
